# Cancer Stem Cells and Epithelial-to-Mesenchymal Transition (EMT)-Phenotypic Cells: Are They Cousins or Twins?

**DOI:** 10.3390/cancers30100716

**Published:** 2011-02-21

**Authors:** Dejuan Kong, Yiwei Li, Zhiwei Wang, Fazlul H. Sarkar

**Affiliations:** Department of Pathology, Karmanos Cancer Institute, Wayne State University School of Medicine, 4100 John R, Detroit, MI 48201, USA; E-Mails: dkong@med.wayne.edu (D.K.); yiweili@med.wayne.edu (Y.L.); zhiweiwang@med.wayne.edu (Z.W.)

**Keywords:** Epithelial-to-Mesenchymal Transition, cancer stem-like cells, tumor-initiating cells, drug resistant

## Abstract

Cancer stem cells (CSCs) are cells within a tumor that possess the capacity to self-renew and maintain tumor-initiating capacity through differentiation into the heterogeneous lineages of cancer cells that comprise the whole tumor. These tumor-initiating cells could provide a resource for cells that cause tumor recurrence after therapy. Although the cell origin of CSCs remains to be fully elucidated, mounting evidence has demonstrated that Epithelial-to-Mesenchymal Transition (EMT), induced by different factors, is associated with tumor aggressiveness and metastasis and these cells share molecular characteristics with CSCs, and thus are often called cancer stem-like cells or tumor-initiating cells. The acquisition of an EMT phenotype is a critical process for switching early stage carcinomas into invasive malignancies, which is often associated with the loss of epithelial differentiation and gain of mesenchymal phenotype. Recent studies have demonstrated that EMT plays a critical role not only in tumor metastasis but also in tumor recurrence and that it is tightly linked with the biology of cancer stem-like cells or cancer-initiating cells. Here we will succinctly summarize the state-of-our-knowledge regarding the molecular similarities between cancer stem-like cells or CSCs and EMT-phenotypic cells that are associated with tumor aggressiveness focusing on solid tumors.

## 1. Introduction

Epithelial-to-Mesenchymal Transition (EMT) was first recognized as a feature of embryogenesis, which is vital for morphogenesis during embryonic development. Recently it has also been implicated in the conversion of early stage tumors into invasive malignancies [1]. Increasing evidence suggests that tumor progression is critically involved with the acquisition of an EMT phenotype, which allows tumor cells to acquire the capacity to infiltrate surrounding tissues, and thus license these cells to metastasize in distant sites [2-4]. Progression of most carcinomas is associated with the acquisition of mesenchymal phenotype, which is accompanied by the loss of epithelial marker expression and up-regulation of mesenchymal molecular markers, leading to increased cell motility and invasion [5]. These processes are consistent with the acquisition of a “cancer stem-like cell” phenotype that is also known as “stemness” or cancer stem cell (CSCs) characteristics [3], although these terminologies are not synonymous. The initiation and recurrence of tumors is believed to be strongly linked with the biology of CSCs or cancer-initiating cells [6-8]. Accumulating evidence have shown that cells with an EMT phenotype induced by different factors are rich sources for cancer stem-like cells [5,9-11], suggesting the biological similarities between CSCs, cancer stem-like cells, cancer-initiating cells and EMT-phenotypic cells. Moreover, induction of EMT in tumor cells not only promotes tumor cell invasion and metastasis but also contributes to drug resistance [12-15], suggesting that the molecular characterization of these cells will allow the development of newer therapies for complete eradication of tumors, which will certainly improve the overall survival of patients diagnosed with cancers.

## 2. The Role of EMT in Cancer Progression and Metastasis

EMT is a process by which epithelial cells undergo remarkable morphological changes characterized by a transition from an epithelial cobblestone phenotype to an elongated fibroblastic phenotype [16]. The process of EMT involves a disassembly of cell-cell junctions [17], actin cytoskeleton reorganization [18] and increased cell motility [1] and invasion [2], as characterized by down-regulation and relocation of E-cadherin and zonula occludens-1 (ZO-1) [19,20] as well as down-regulation and translocation of β-catenin from the cell membrane to nucleus, and up-regulation of mesenchymal molecular markers such as vimentin [18, 21], fibronectin and N-cadherin [1,4,12,16]. During the processes of EMT, non-motile epithelial cells with regular cell-cell junctions and adhesion, lose their cell-cell junctions and convert into individual, motile and invasive mesenchymal phenotypic cells. The idea that EMT is relevant in cancer was initially met with skepticism because pathologists could not find conclusive evidence in support of the presence of EMT in human tumor samples [22-25]. However, increasing evidence have demonstrated that the process of EMT is vitally important in cancer progression and metastasis [2,3,5,12,22,26-29]. Progression of solid tumors occurs through a spatial and temporal emergence of EMT, thereby the tumor cells acquire a more invasive and metastatic phenotype. Metastatic tumor cells with a mesenchymal phenotype are believed to undergo reverse transition, *i.e.*, Mesenchymal-to-Epithelial Transition (MET) at the site of metastasis to gain the pathology of their corresponding primary tumors [12,22]. This process is a critical step by which metastatic tumor cells grow at the secondary site. Recent studies have shown that primary colon carcinomas and their corresponding metastasis exhibited a mixed epithelial-mesenchymal phenotype [2]. Cells in the tumor center remain positive for the expression of E-cadherin and cytoplasmic β-catenin, and the tumor cells in the periphery display loss of surface E-cadherin and up-regulation of vimentin as well as nuclear β-catenin staining, the typical characteristics of EMT phenotype [2,22]. More importantly, Chaffer *et al.* used bladder carcinoma TSU-pr1 (T24) series of cell lines selected *in vivo* for increasing metastatic ability following seeding through systemic circulation, and found that the more metastatic sub-lines had acquired EMT characteristics [27]. In prostate cancer, Yates *et al.* performed co-culture of hepatocytes and DU145 or PC3 cells and found that DU145 and PC3 cells displayed E-cadherin up-regulation at peripheral sites of contact under the co-culture conditions [30]. Although the PC3 cell line is a highly malignant prostate cancer cell line derived from metastatic tumors to the bone, the majority of EMT studies in prostate cancer have used PC3 cells [12]. These cell lines showed expression of molecular markers of cell-cell adhesion junctions such as E-cadherin concomitant with epithelial-like morphology, which is consistent with the characteristics of primary epithelial tumor cells. It is highly likely that prostate cancer cells from the primary site in patients undergo EMT may have also acquired MET characteristics when they arrive at the site of metastasis (such as bone and the brain from where PC3 and DU145 cells, respectively, were originally derived). This could also be associated with the acquisition of an incomplete epithelial phenotype or mixed phenotype, typically known as fused cell phenotype [12,22]. Emerging evidence suggests that the process of EMT is triggered by a molecular interplay between extra-cellular signals such as collagen and growth factors including transforming growth factor-β (TGF-β), fibroblast growth factor (FGF), epidermal growth factor (EGF) and platelet-derived growth factor (PDGF) A, B and D [31-35]. In a recent study, Graham *et al.* showed that IGF-1 could activate an EMT phenotype in PC3 cells, which was mediated by the activation of ZEB1 (zinc finger E-box binding homeobox 1) [36]. These results suggest that EMT phenotypic changes in cells contribute to tumor aggressiveness.

## 3. Cancer Stem Cells (CSCs) or Cancer Stem-Like Cells

The cancer stem cells (CSCs) are cells within a tumor that possess the capacity to self-renew and differentiate into the heterogeneous lineages of cancer cells that comprise the whole tumor. These tumor-initiating cells could provide a reservoir of cells that cause tumor recurrence after therapy. The existence of CSCs was first identified by Dick and coworkers in leukemic cells [37]. They found that only a minor subset of leukemic cells with the CD34^+^CD38^−^ cell surface marker profile was transplanted into severe combined immune-deficient (SCID) mice, resulting in a pattern of dissemination and leukaemic cell morphology similar to that seen in the original patient [37]. Recently, CSCs have been identified in solid tumors such as breast, colon, brain tumors and prostate cancer [38-43], Ricci-Vitiani *et al.* found that 10^5^ CD133^−^ colon cancer cells did not induce tumor formation. The injection of 10^6^ total colon cancer cells resuspended in matrigel generated visible tumors after five weeks, whereas injection of 3,000 CD133^+^ cells induced visible tumors after four weeks [40]. O'Brien *et al.* also found that as few as 262 CD133^+^ colon cancer cells could induce tumor formation in severe combined immune-deficient (SCID) mice [39]. There results indicated that colon-cancer initiating cells are CD133^+^ colon cancer cells. Singh *et al.* isolated the brain tumor stem cells (BTSC), the subset with the increased self-renewal capacity was derived from the most aggressive clinical samples of medulloblastomas compared with low-grade gliomas by using the neural stem cell surface marker CD133 [41,42]. These CD133^+^ cells could differentiate in culture into tumor cells that phenotypically resembled the tumor from the patient. They also demonstrated that injection of as few as 100 CD133^+^ cells induced tumor initiation in NOD-SCID (non-obese diabetic, severe combined immunodeficient) mouse brains. In human prostate cancer, Patrawala *et al.* identified tumor-initiating cells from established xenografts by using the CD44 surface marker and enriched these cells by sorting CD44^+^α2β1^+^ cells [44]. Since the majority of the human PCa possess the mature luminal phenotype cells characterized by the expression of cytokeratin 8/18, androgen receptor (AR) and prostate specific antigen (PSA), the hypothesis has been that the cell of origin of PCa is a differentiated luminal cell. However, there is high phenotypic heterogeneity within PCa, including metastatic sites, containing rare cells that are phenotypically undifferentiated [45]. Although cell of origin of PCa needs to be fully elucidated, mounting evidence demonstrates that tumor-initiating cells play a critical role in the progression and recurrence of PCa [6-8,46,47]. Recent studies indicated that co-expression of pluripotency markers such as Oct4, Sox2, Nanog, lin28, Klf4 and c-myc can reprogram somatic cells into pluripotent embryonic stem-like cells [48-50], suggesting that combined expression of stem cell-associated factors in cells with oncogenes could also induce an undifferentiated state in these cells. Interestingly, Gu *et al.* found that cell lines derived from human prostate specimens with epithelial phenotype were immortalized by hTERT and showed expression of embryonic stem cell markers such as Oct4, Nanog, and Sox2 [46], which is consistent with the results showing that over-expression of Oct4, Sox2, Nanog and c-myc has been found in poorly differentiated tumors [51]. Nanog, Sox2 and Oct4 have been shown to play important roles in the progression of cancer [52-55]. Most interestingly, increasing evidence suggests that EMT induced by different factors is associated with metastasis and also associated with the generation of stem-like cells [5,9-11, 47].

## 4. EMT-Phenotypic Cells as a Resource for CSCs

Progression of most carcinomas toward malignancy is associated with the loss of epithelial differentiation and gain of mesenchymal phenotype as characterized by increased cell motility and invasion [1], resulting in tumor metastasis [5] and drug resistance [15]. These processes are believed to be associated with EMT [1,16,56,57]. Recent studies have demonstrated that EMT plays a critical role not only in tumor metastasis but also in tumor recurrence, which is tightly linked with the biology of CSCs [14,58-64]. Morel *et al.* demonstrated that CD44^+^CD24^−/low^ stem-like cell signatures could be generated from CD44^low^CD24^+^cells, non-tumorigenic mammary epithelial cells, through activation of the Ras/MAPK signaling pathway. Further, they also found that CD44^+^CD24^−/low^ cells displayed an EMT phenotype as characterized by the loss of E-cadherin expression and gain of vimentin expression. They hypothesized that the induction of EMT could be responsible for switching CD44^low^CD24^+^ cells to CD44^+^CD24^−/low^ stem-like cells. To this end, CD24^+^ cells treated with TGF-β, a potential inducer of EMT, led to CD24 cell appearance eight days after treatment, concomitant with enrichment of mesenchymal phenotypic cells as characterized by the loss of E-cadherin and the gain of vimentin expression [65]. Mani *et al.* further demonstrated that the induction of non-tumorigenic, immortalized human mammary epithelial cells into EMT phenotype induced by the expression of either twist or snail, well known transcription repressors, resulted in the loss of epithelial phenotype and the acquisition of mesenchymal phenotype concomitant with the acquisition of CD44^high^/CD24^low^ expression pattern and increased mammosphere-forming ability as well as tumor initiating capacity [9]. Whereas, isolated CD44^high^ /CD24^low^ stem-like cells from normal and neoplastic human mammary cells exhibited a mesenchymal morphology and expressed mesenchymal markers such as vimentin and fibronectin [9]. Santisteban *et al.* observed that the induction of EMT by an immune response against an epithelial breast cancer led to the outgrowth of tumor *in vivo* [11]. Interestingly, the resulting mesenchymal tumor cells had a CD44^+^CD24^−/low^ phenotype with the ability to reestablish an epithelial tumor and increased drug resistance, which is consistent with breast CSCs [11]. More recently, Gupta *et al.* also found that the induction of EMT in transformed HMLER breast cancer cells by shRNA-mediated knock-down of E-cadherin expression displayed an increased population of CD44^high^ /CD24^low^ cells, and these cells exhibited a ∼100-fold enhanced mammosphere-forming ability compared to their epithelial phenotypic cells [66]. More importantly, they found that EMT cells displayed an increased drug resistance associated with CSCs signatures [66]. These reports strongly suggest that the induction of EMT could generate stem-like cells; however, the molecular mechanisms responsible for such processes are not fully understood.

## 5. The miRNAs linking EMT with Stem Cell Signatures

It is known that microRNAs (miRNAs) are involved during embryonic development and in cancer progression [67], a process that is known to be associated with the acquisition of EMT phenotype of epithelial tumor cells [68]. The miRNAs are small (19-24 nucleotides) non-coding RNA molecules which down-regulate gene expression by interacting with seed sequences located in the 3′UTR of multiple target mRNAs, resulting in either translational repression or degradation of mRNAs [69]. The evolutionarily conserved family miR-200 has been implicated in regulation of the differentiation processes during development [68]. Recent studies have also shown that miR-200 family members could regulate the processes of EMT by regulating ZEB1 and ZEB2 expression through binding to the sequences at the 3′UTR of ZEB1, ZEB2 mRNA [10,19,70-73]. ZEB1 and ZEB2 could repress the expression of miR-200 family by directly binding to E-box binding sites in the promoter of the miR-200 gene cluster, establishing a double negative feedback loop controlling ZEB1, ZEB2 and miR-200 family expression during EMT [74]. Furthermore, miR-200 has also been shown to be associated with stem-like cell signatures by regulating the expression of Bmi1, Notch1 and Lin28B expression [75-77]. Shimono *et al.* found that the miR-200 family was strongly suppressed in CD44^+^CD24^−/low^ lineage human breast cancer cells and normal human mammary stem cells, whereas miR-200c strongly suppressed the ability of normal mammary stem cells to form mammary ducts and tumor formation driven by human breast CSCs *in vivo* [76]. They also found that miR-200c repressed the expression of Bmi1, which is associated with the regulation of stem cell self-renewal [76]. Wellner *et al.* showed that the EMT-activator ZEB1 was strongly expressed in less differentiated human pancreatic cancer, and orthotopic (intrapancreatic) injection of Panc1 cells with ZEB1 expression resulted in the formation of a large primary tumor invading into stomach, spleen, small and large bowel, and metastasizing to lymph nodes as well as the liver in nude mice. In contrast, injection of cells with knock-down of ZEB1 resulted in smaller primary tumors with almost no local infiltration and without lymph nodes and distant metastasis. More importantly, they have also demonstrated that ZEB1 is necessary for tumor-initiating capacity of pancreatic and colorectal cancer cells. They found that ZEB1 not only repressed the expression of miR-200c but also controlled the expression of “stemness” associated factors such as Bmi1, Sox2 and Klf4 by inhibiting miR-203 and miR-183 expression [77]. Therefore, ZEB1 could be a promising target for the treatment of tumors. More recently, Yang *et al.* revealed that Bmi1 played an essential role in Twist1-induced EMT of head and neck squamous cell carcinoma, and that the ectopic expression of Twist1 not only increased Bmi1 expression but also induced the stem cell marker Sox2 expression. They further showed that Twist1 directly regulated the expression of Bmi1. Twist1 and Bmi1 were mutually essential to promote EMT and tumor-initiating capacity by up-regulating stem cell factors and by repressing the expression of both E-cadherin and p16INK4a [63]. These reports strongly suggest that the miR-200 family is directly linked with the regulation of EMT and the maintenance of CSCs and stem-like cell characteristics.

Among many signaling pathways, Akt is known to play a critical role in human cancer initiation and progression, and it is also associated with the induction of EMT phenotype [28]. Interestingly, Iliopoulos *et al.* demonstrated that three isoforms of Akt played contrasting roles in the induction of EMT by regulating the expression of the miR-200 family. They expressed each isoform individually in an Akt-1^−/−^/Akt-2^−/−^/Akt-3^−/−^ cell line and found that the expression of the miR-200 family was significantly decreased in cells expressing Akt-2 [78]. Knock-down of Akt-1 in transforming growth factor-β (TGF-β)-treated MCF-10A cells also decreased the expression of miR-200 and promoted TGF-β-induced EMT as characterized by decreased expression of E-cadherin, and induced stem-like cell phenotype by increasing mammosphere-forming ability. Concomitantly, carcinomas developing in MMTV-cErbB2/Akt1^−/−^ mice showed down-regulation of miR-200 and increased invasiveness. Therefore, the ratio of Akt-1 and Akt-2 rather than the overall activity of Akt could control the induction of EMT and maintenance of “stemness” by regulating the expression of the miR-200 family [78]. Recently, they also found that the miR-200 family was inhibited during cancer stem cell induction but not transformation in an MCF-10A model carrying an inducible Src oncogene (ER-Src), and inhibition of miR-200b showed increased CSC formation. Interestingly, they demonstrated that miR-200b directly targeted Suz12, a subunit of a polycomb repressor complex (PRC2) [79]. PRC2 contains Suz12, EZH2, EED and RbAp subunits and is known to be involved in the regulation of gene repression through chromatin modifications, which is essential for the maintenance of embryonic and adult stem cells [80,81]. PRC2 mediated repression of the E-cadherin gene promoted induction of EMT. Moreover, further studies have shown that PRC2 target genes are co-occupied by stem cell regulators such as Oct4, Sox2 and Nanog [80-82]. Ilipoulos *et al.* found that the loss of miR-200 during CSC formation could increase Suz12 expression, and re-expression of miR-200b or Suz12 depletion blocked the formation and maintenance of mammospheres [79]. Conversely, ectopic expression of Suz12 in transformed cells promoted the generation of CSCs [79]. These results suggest that the miR-200b-Suz12-E-cadherin pathway is involved in CSCs maintenance and invasive characteristics of breast cancer cells.

We have recently found that platelet-derived growth factor-D (PDGF-D), a newly recognized growth factor, which is known to regulate many cellular processes including cell proliferation, transformation, invasion and angiogenesis, induced EMT in PC3 PCa cell line by down-regulating the expression of the miR-200 family, resulting in increased expression of ZEB1, ZEB2 and slug [20,70,75]. The expression of miR-200 was significantly reduced in PC3 cells exposed to purified active PDGF-D protein compared to parental PC3 cells, which was associated with the over-expression of ZEB2 and slug. Interestingly, re-expression of miR-200 in PDGF-D over-expressing PC3 cells with EMT phenotype led to the down-regulation of ZEB1, ZEB2 and slug with corresponding up-regulation of epithelial markers such as E-cadherin, stratifin, EpCAM, F11R and connexin 26, and decreased expression of vimentin [70]. From these results, we concluded that the loss of miR-200 plays an important role during the acquisition of EMT phenotype of PC3 cells induced by PDGF-D, and that the re-expression of miR-200 could cause the reversal of the EMT phenotype to MET phenotype. Interestingly, we also found that the EMT-phenotypic PC3 cells induced by over-expression of PDGF-D shared stem-like cell features as characterized by enhanced clonogenicity, self-renewal capacity as well as increased tumorigenicity in mice, which was consistent with increased expression of stem cell markers such as Notch-1, Sox2, Nanog, Oct4 and Lin28B. These EMT-type cells also showed decreased expression of the miR-200 or let-7 family. More importantly, reversal of EMT by forced re-expression of miR-200 by transfection of miR-200 precursors significantly inhibited clonogenic and prostasphere-forming ability, which was associated with the down-regulation of Notch-1 and Lin28B expression [75]. Moreover, knock-down of Lin28B markedly increased let-7 expression and reduced self-renewal ability. Concomitantly, we also found that ARCaP_M_ cells with EMT phenotype also shared stem-like cell signatures consistent with increased expression of Notch-1 and enhanced clonogenic and prostasphere-forming ability compared with control cells (ARCaP_E_ cells) with epithelial phenotype. The miR-200c was repressed in ARCaP_M_ cells with EMT phenotype and the re-expression of miR-200c reversed EMT phenotype to MET phenotype associated with down-regulation of Notch-1 expression and self-renewal capacity of ARCaP_M_ cells [75] These reports strongly suggest that miRNAs, especially miR-200 family members, link EMT phenotype with stem cell signatures (Table 1).

**Figure 1.**
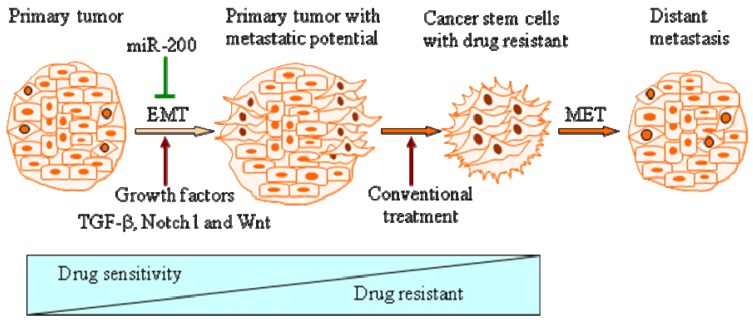
Induction of epithelial-to-mesenchymal transition (EMT)-phenotypic cells produces cancer stem-like cells with drug-resistant characteristics. Growth factors, including FGF, EGF, PDGF-B and PDGF-D as well as factors such as TGF-β, Notch-1 and Wnt, can induce EMT, while miR-200 family inhibits EMT by regulating the expression of transcription repressors ZEB1 and ZEB2. EMT-phenotypic cells acquire stem-like cell signatures characterized by increased metastatic capacity, self-renewal ability and acquired drug resistance. These cells metastasize to distant sites and undergo MET to produce metastatic tumors that are phenotypically similar to the primary tumor.

Mounting evidence has shown that induction of EMT by different factors could generate stem-like cells characterized by enhanced self-renewal and invasive capacity and high drug resistance, which is strongly associated with metastases and recurrence of tumors (Figure 1).

## 6. Perspectives

Conventional treatment for cancers mainly targets the differentiated tumor cells; however, in a significant number of patients, cancer cells will acquire a drug resistant phenotype after standard therapies, resulting in tumor recurrence and metastasis for which there is limited or no curative therapy. The recurrence of tumors is believed to be tightly linked with the biology of CSCs or cancer-initiating cells [6-8]. Mounting evidence has demonstrated that the acquisition of invasive characteristics of tumors is also associated with the ability of tumor cells to undergo EMT phenotype, which allows tumor cells to break through the structural constraints imposed by tissue architecture [3,4,12,22]. The stem-like cells or CSCs generated from EMT induction provide a resource for cancer to recur and these cells are well known to be highly drug resistant [9,15,65,67,72]. Therefore, it is important to identify which factors could induce EMT and uncover the mechanistic role of such factors during cancer progression, which underscores the importance of such factors toward the development of novel and targeted therapies for complete eradication of cancer. The molecular understanding and the biological characteristics of CSCs, cancer stem-like cells and EMT phenotypic cells will allow us to screen for potential drugs that could cause selective killing of these cells to eradicate tumor recurrence. Moreover, agents that may result in the re-expression of specific miRNAs that are lost in these cells will also allow us to eliminate the cells that are the “root cause” of tumor development, maintenance, recurrence and metastasis. Thus, the future appears to be brighter than ever before for complete eradication of cancer by exploiting current molecular understanding of CSCs, and the processes of EMT.

## Figures and Tables

**Table 1.** miRNAs linking epithelial-to-mesenchymal transition (EMT) phenotype with stem-like cell signatures in human cancers.

**miRNAs**	**Functions in Regulation of EMT and Stem Cell Signatures**	**Ref.**
miR-200a	knockdown of Akt-1 decreases expression of miR-200 family including miR-200a, increases mammosphere forming ability in breast cancer	[78]
miR-200b	miR-200b inhibits expression of ZEB1, ZEB2, Lin28B and Notch1 in prostate cancer miR-200b targets Suz12 and contributes to maintain cancer stem cells in breast cancer	[75,79]
miR-200c	miR-200c inhibits expression of ZEB1, ZEB2 and Bmi1 in breast cancer; miR-200c also inhibits expression of ZEB1, Sox2, Bmi1 and KLF4 in pancreatic cancer	[77]
miR-183	miR-183 downregulated by ZEB1 and inhibits expression of Bmi1 and KLF4 in pancreatic cancer	[77]
miR-203	miR-203 downregulated by ZEB1 and inhibits expression of Bmi1 and KLF4 in pancreatic cancer	[77]
